# Filter Transmittance Measurements in the Infrared

**DOI:** 10.6028/jres.098.045

**Published:** 1993

**Authors:** A. L. Migdall, A. Frenkel, D. E. Kelleher

**Affiliations:** National Institute of Standards and Technology, Gaithersburg, MD 20899-0001

**Keywords:** attenuators, direct detection, filter transmittance, heterodyne detection, infrared

## Abstract

We have set up a novel direct detection system to measure filter transmittances over an attenuation range of at least 5 decades, with relative combined standard uncertainties as low as 0.5% (1*σ*) per decade, in the 9 *μ*m to 11 *μ*m spectral region. This system, using an apparatus originally designed for a heterodyne measurement of transmittance, achieves higher accuracy at the expense of a reduced dynamic range. This independent measurement of transmittance allows verification of the heterodyne technique. Our system uses a source modulated at 30 MHz and a specially constructed high dynamic range and high accuracy lock-in amplifier capable of operation at the modulation frequency. The high modulation frequency and narrow bandwidth of the system allow thermal background radiation to be suppressed and high accuracy to be achieved. We correct for the non-ideal natures of the detector and attenuators. In particular, the detector position is scanned to reduce the effect of its spatial nonuniformity and the deflection of the transmitted beam caused by the nonparallel surfaces of the filter. We discuss the sources of systematic errors and the methodology to reduce their contribution.

## 1. Introduction

We have developed a method for accurately measuring a wide range of filter attenuation in the infrared (ir) using a direct detection system. The method makes use of a specially constructed high frequency lock-in amplifier which allows ir attenuation measurements to be tied to a high accuracy radio frequency attenuation standard. We have measured the absolute attenuation of filters at two wavelengths, 10.2 *μ*m and 10.6 *μ*m. Subsequent measurements will use an ir Fourier Transform Interferometer to determine the spectral variation of the attenuation of these filters. Ultimately this work will allow the National Institute of Standards and Technology to provide calibrated neutral density filters over a wide range of attenuations.

The measurement scheme described here is a high accuracy direct detection method with a dynamic range of more than 5 decades. This system was set up to allow independent verification of an even higher dynamic range heterodyne detection scheme described elsewhere [1,2]. The filters measured here, along with the estimated uncertainties, will be used to corroborate the accuracy of the higher dynamic range heterodyne detection scheme.

Accurate methods for measuring filter attenuations are well established in the visible region of the spectrum. This is not the case in the infrared however, where measurements become increasingly difficult, particularly at large attenuations. The problems are associated with both detectors and attenuators. Detectors in the ir generally have inferior spatial uniformity and are linear over restricted dynamic ranges, compared to visible detectors, while attenuators in the ir are not neutral because bulk materials with spectrally neutral absorption are unavailable. As a result most ir attenuators are reflective coatings on transmitting substrates or bulk absorbing cutoff filters. One disadvantage of reflective attenuators is the interference effect that becomes evident when used with laser sources [3]. This is a particular problem with highly attenuating filters whose optical density (*OD* = −log_10_*T*, where *T* is the filter transmittance) is difficult to measure with a thermal source. Finally, the temperature dependence of both detectors and filters is generally worse in the ir.

## 2. Experimental Setup

Our measurement scheme uses a CO_2_ laser modulated at 30 MHz, a high frequency detector and lock-in amplifier. The ratio of detected signals with and without the filter in the laser beam is used to determine the filter *OD.* The high frequency of modulation reduces laser noise common at low frequencies and allows the use of a high accuracy and high dynamic range lock-in amplifier. The narrow bandwidth capability of the lock-in amplifier allows us to neglect the ambient thermal background radiation which peaks in the CO_2_ laser wavelength range. The particular modulation frequency, 30 MHz, was chosen because there exists a radio frequency (rf) attenuation standard at that frequency at NIST. That makes possible a lock-in amplifier system capable of high accuracy amplitude measurement as well as high dynamic range, as opposed to lock-in systems where only dynamic range or frequency selectivity is the goal.

**Fig. 1:**
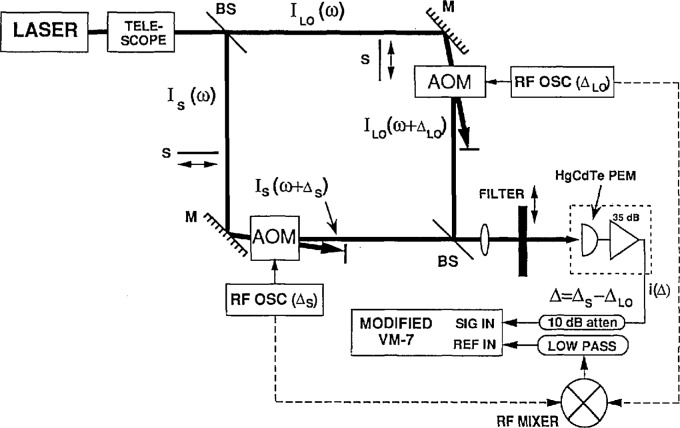
Experimental setup – mirrors, beamsplitters and shutters are labeled M, BS, and S, respectively.

The layout of the experimental setup is shown in Fig. 1. The source is a grating tuned frequency-stabilized CO_2_ laser. The frequency of the laser is locked to the center of a line determined by the grating. This stabilizes the laser intensity to better than 0.5% maximum deviation over an hour and to within 4% over a day. After passing through an adjustable attenuator, the output of the CO_2_ laser is split into two beams. Each of these beams is sent through an acousto-optic modulator (AOM). One of the AOMs is set to upshift the frequency of the beam passing through it by 40 MHz, while the other AOM upshifts its beam by 70 MHz. These two frequency shifted beams are then recombined and made to pass through a lens, an aperture and the mounted filter before final detection. The combined beams induce a signal at 30 MHz, the difference frequency. The dual beam frequency shifts allow a 30 MHz modulation of the ir signal to be generated without requiring a 30 MHz driver for an AOM. This eliminates the need to shield the ir detector from the AOM driver, which uses ~ 10 W of rf power. Shutters are used for baseline subtraction to eliminate the effects of any internal lock-in offsets or any coherent pickup.

The 30 MHz amplitude is measured using a lock-in amplifier based on two custom-modified 30 MHz Weinschel VM-7^1^ attenuator and signal calibrators [2,4]. The original instruments were designed for 30 MHz attenuator calibrations. The modifications allow the instrument to function as a true dual phase lock-in amplifier at 30 MHz while retaining the high accuracy of the original instrument.

Our tests on the present form of the modified instrument show a dynamic range of about 180 dB. The manufacturer’s specifications for the accuracy of the original instrument were 0.02 dB per 10 dB from 0 dBm to −100 dBm, 0.04 dB per 10 dB from −100 dBm to −110 dBm and 0.12 dB per 10 dB from −110 dBm to −120 dBm. Our own linearity tests of the modified instrument [2] indicate that the standard uncertainty [5] is ~1/3 of these values.

We use a temperature stabilized photoelectro-magnetic (PEM) HgCdTe detector with a 1 mm × 1 mm active area. The thermal time constant is on the order of minutes, so the thermal regulation cannot respond to rapid effects such as heating by the laser as the filter is moved in and out of the beam. However, as described later, tests of the linearity of the entire system showed that this is not a problem for all but the highest laser powers. The detector is mounted on a two axis motorized positioning stage allowing the detector to be centered with respect to the optical beam. This is important due to detector nonuniformity and deflection of the transmitted beam by filters with nonparallel surfaces. The detector is followed by an rf preamplifier having a gain of 35 dB, and a 10 dB rf attenuator which we found necessary to limit oscillations caused by interaction between the preamplifier and lock-in amplifier.

The filter type itself is crucial to the ultimate accuracy of these measurements. We investigated two types of filters for this project: filters made from a bulk absorbing material and the more usual reflective coating on a substrate. We discuss later the merits of both filter types and how their characteristics affect the transmittance accuracy.

The absorptive type attenuator samples were made of three different thicknesses of LiF. This material is highly absorbing at wavelengths beyond about 9 *μ*m. Since the filter absorption is proportional to thickness, special care was taken to make the thicknesses uniform across the samples. We were able to make the samples flat to 10 *μ*m across the 25 mm samples. The observed deflection of an 633 nm beam through the filter was less than 0.15 mrad. The reflective filters tested were commercially available neutral density filters with metallic films on germanium substrates. Both types of filters were held in a temperature stabilized copper block. Two thermistors were mounted in holes in the block for monitoring and controlling the temperature.

## 3. Measurement Technique

The measurement procedure was designed to minimize uncertainties due to the nonideal nature of the detectors and filters. Detectors in the ir generally have poor spatial uniformity. The uniformity of the PEM HgCdTe detector used in our work was found to vary by a factor of 3 from the center to the edge, so it is clearly important to recenter a beam that is slightly deflected as it passes through a filter that is not perfectly parallel. To accomplish this, the detector was scanned through a 4 × 4 x-y grid of 16 points. These scans were taken for both the filter in and filter out positions. To determine the peak signal, or optimal centering, each set of 16 points was fit to a two-dimensional parabolic surface. The transmittance was found from the ratio of the fitted peak signals with the filter in and filter out. Zero levels measured with the shutters closed were subtracted before fitting the data. The ratio of optical beam size to detector diameter was adjusted by moving the detector through the beam focus to minimize the rms deviation between the parabolic fit and the data. This was done to minimize the sensitivity of the measurement to spatial variations of the beam profile and detector uniformity. The optimum was reached when the beam size was on the order of the detector diameter.

For the absorptive type filters this was all that was required to extract an optical density from the data. This advantage in ease of measurement must be weighed against the steep wavelength dependence of the absorption. The reflective type filters however, while spectrally uniform when used with broadband sources, require a more complicated measurement procedure. This is because the use of a narrow band light source, such as a laser, with a reflective type attenuator introduces complications that do not occur with a broadband source or bulk absorbing attenuator. To the extent that a filter substrate has flat and parallel surfaces, it will act as a Fabry-Perot etalon. (Producing a filter that is not parallel will reduce this effect, but will also deflect the transmitted beam which causes other complications.) The overall visibility of the resulting fringes varies as the geometric mean of the reflectances of the front and back surfaces. Even if care is taken to put a good antireflection (0.1% reflectivity) coating on the second surface, the geometric mean of the two reflectivities is large enough to produce significant interference effects (±0.03 *OD).* (In addition, the spectral dependence of antireflection coatings can reduce the useful wavelength range of the filter.) This results in a transmittance that varies periodically with laser wavelength. The period of this variation (*λ*^2^/2*nL* where λ is the wavelenght, *n* is the refractive index of the substrate and *L* is the filter thickness) is about 0.01 *μ*m for a 1 mm thick substrate.

To obtain a reflective filter transmittance that can be compared with a transmittance measured using a broadband source, we must average over the variations with laser wavelength. This could be accomplished by scanning the laser wavelength, but our laser is not capable of scanning continuously over a large enough range. We show preliminary results of an alternate method to allow the transmittance variation to be observed without varying the laser wavelength. This is done by using temperature to vary the effective thickness, or optical path length, of the filter. This is possible because the index of refraction of the substrate is temperature dependent. To minimize the temperature variation required, we chose Ge as a substrate material, because its index temperature coefficient is extremely large, 396 × 10^−6^/°C. With this material, a temperature range of 12 °C allows a complete period of the transmittance variation to be mapped.

Since the filter thickness varys across its aperture and the laser beam divergence is not zero, the actual shape of the transmittance variation is the Fabry-Perot function integrated over these variations. For simplicity, we fit the transmittance variation versus temperature to a sine function plus an offset term, *A + B* sin(*C_t_ + D*), where *A, B, C*, and *D* are constants and *t* is the temperature. The offset term, *A*, is what we used as the average transmittance. Because the amplitude of the *OD* variation was not too large (typically ±0.01 to ±0.05), this procedure allows us to extract an average transmittance with reasonable accuracy even though the sinusoidal form is not quite the right shape. The ultimate accuracy of this method will require further investigation.

## 4. Results

We measured the transmittances of three thicknesses of LiF at two wavelengths, 10.2 *μ*m and 10.6 *μ*m and at several temperatures. The results measured at a temperature of 29.6 °C are shown in Table 1 with the last columns showing the temperature dependence of the attenuation at the two wavelengths.

**Fig. 2:**
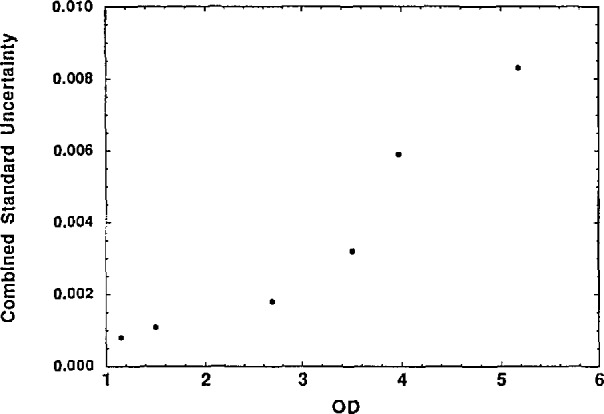
*OD* combined standard uncertainty vs *OD* for the LiF filter measurements.

The combined standard uncertainties of the *OD* values are mainly dominated by the VM7 measurement uncertainty. The results, as shown in Fig. 2, can be approximated by an overall value of 0.002 *OD*/*OD* or about 0.5% transmittance uncertainty per decade. The make up of the total combined standard uncertainties of the *OD/L* values are the quadrature sum of the standard uncertainties of the sample temperature (~20 mK) and thickness, wavelength (assuming 3 × 10^−5^ *μ*m laser linewidth), statistical and VM7 measurement uncertainties. Of these, sample thickness and the VM7 are the two major sources of uncertainty.

The thickness uncertainty was dominant for small *OD* samples, while the VM7 uncertainty was dominant at large *OD.* The sample thicknesses were measured by the Precision Engineering Division at NIST using an electro-mechanical dual probe comparator referenced to NIST gauge blocks. The standard uncertainty with which the center thickness could be determined was 0.17 *μ*m. The major component of this uncertainty was due to variations of the sample thickness, which were as great as 3.3 *μ*m across the central 50% of the 25 mm filter diameter. Since the optical beam diameter was a few millimeters and the filter position relative to the beam varied somewhat for these measurements, we estimate the standard uncertainty of the thickness to be *~*2 *μ*m. The values of *OD*/*L* are for the bulk sample material with the estimated surface reflectance subtracted off before calculating the attenuation per thickness. The calculated component of optical density due to the reflectance of two surfaces is 0.00158 at 10.2 *μ*m and 0.00068 at 10.6 *μ*m with a standard uncertainty of 0.0002.

These results with LiF filters demonstrate a dynamic range of the technique of over 5 decades. The two different wavelength measurements of *OD*/*L* for the 0.7068 mm and 2.4308 mm samples agree to within the uncertainties. The *OD*/*L* of the 1.6200 mm sample differs from the other two by amounts well beyond the uncertainties. The ratio of the attenuation per thickness of the 1.6200 mm sample to the average of the other two samples is 1.0163 and 1.0171 at 10.2 *μ*m and 10.6 *μ*m, respectively. The consistency of this difference (and the fact that the 0.7068 mm and 2.4308 mm samples were ordered from one source while the 1.6200 sample came from another vendor) suggests that it is due to a material variation rather than a measurement uncertainty.

We are able to compare the results here with measurements of LiF using the heterodyne measurement method published earlier [2]. The 1.6200 mm sample measured here, was in fact made from the 2.06 mm sample measured in Ref. [2]. The heterodyne optical density measurement result of 2.120 mm^−1^ from the 2.06 mm sample was taken at 10.59 *μ*m and at an estimated temperature of 24.8 °C. The value obtained here for the 1.6200 mm sample adjusted to 10.59 *μ*m and 24.8 °C is 2.1154 mm^−1^. The difference between these two measurements is about the same as the combined standard uncertainty of the direct measurement. This is reasonable agreement, considering that the heterodyne result was made to test dynamic range rather than accuracy. In particular, the reason for reworking the original 2.06 mm sample was that its optical flatness and parallelism were deficient enough to noticeably deflect and distort a visible laser beam. The effect of this defect on the heterodyne result was not corrected for.

The results here can also be compared with the results of Hohls [6]. By interpolation between stated wavelengths and adjusting for the temperature difference we obtain a value of 1.681 mm^−1^ and 2.128 mm^−1^ for 10.2 *μ*m and 10.6 *μ*m, respectively. As the estimated combined standard uncertainty of this early data is 5%, these values are in good agreement with our results.

**Fig. 3:**
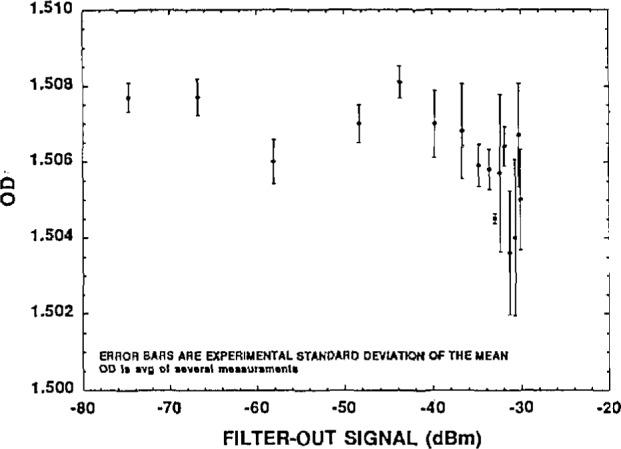
*OD* vs signal for LF sample. Demonstrates measurement linearity vs laser power.

These results, consistent measurements of *OD*/*L* for different thicknesses, put an upper limit of ~0.002 *OD*/*OD* on any nonlinearity error in the measurement system. In addition, we checked linearity by measuring the *OD* of a single LiF filter at a range of laser powers. The results (Fig. 3) show the measured *OD* as a function of signal seen by the lock-in amplifier with filter out of the optical path. These signals correspond to laser powers between about 0.2 mW and 200 mW at the LiF filter. The *OD* is nearly independent of signal level, except at the high end, where there may be a hint of saturation at about the 0.001 level. All our measurements were taken with lock-in input signals below −30 dBm.

**Fig. 4:**
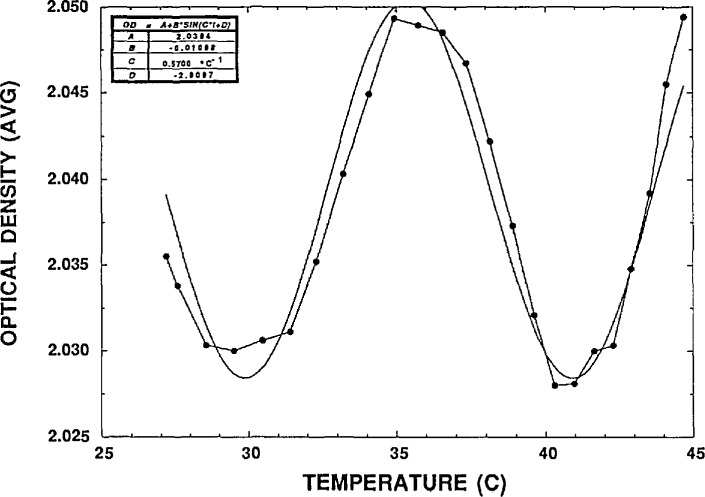
*OD* vs temperature and fit for reflective filter.

The measurements using reflective filters were more difficult. Figure 4 shows the *OD* of a sample filter versus temperature. The variation due to interference is clearly visible. The fit function and parameters are also shown. Although the systematic deviation between the fit function and the data is clearly noticeable, we estimate that the offset parameter *A* is determined to within 0.01. The repeatability of these measurement results was significantly better than this fit determination. While this procedure allows the interference effects associated with reflective filters to be observed, it will require more work to assign a combined standard uncertainty to the measurements. We need to be able to characterize the divergence and uniformity of the laser wavefront as well as the wavefront distortion of the filter itself. For some reflective filters tested, we were able to observe the fringe pattern in the back reflected beam. We saw about 10 straight fringes indicating a wedged filter that did not greatly distort the wavefront.

## 5. Conclusions

We have demonstrated a direct detection scheme capable of measuring optical densities of filters over a dynamic range of 5 decades. The relative combined standard uncertainty appears to be about 0.002 *OD*/*OD* as demonstrated using a bulk absorbing material. For reflective type filters, the accuracy with which the high spectral resolution laser measurements can be averaged over to obtain a low resolution (or broadband) result must be studied further. Other future work will include measurements of transmittance at cryogenic temperatures, wide spectral range FTIR measurements of transmittance, development of NIST Standard Reference Material attenuation filters for the ir and measurements of *OD’*s ranging from about 2–10 using the heterodyne detection method.

## Figures and Tables

**Table 1:** Measured attenuation of LiF filter samples at two wavelengths

Wavelength (*μ*m)	Sample thickness *L* (mm)	*OD*	*δ*/*OD* 1*σ*	*OD*/*L* (mm^−1^)	*δ*(*OD*/*L*) 1*σ* (mm^−1^)	*OD*/(*Lt*) (mm^−1^ °C^−1^)	*δOD*/(*Lt*) 1*σ* (mm^−1^ °C^−1^)
10.2	0.7068	1.155	0.0008	1.632	0.0048		
	1.6200	2.690	0.0018	1.659	0.0023	0.0044	0.0002
	2.4308	3.971	0.0059	1.633	0.0028		
10.6	0.7068	1.507	0.0011	2.132	0.0062		
	0.6200	3.511	0.0032	2.167	0.0033	0.0063	0.0006
	2.4308	5.175	0.0083	2.129	0.0038		

## Footnotes

1 Certain trade names and company products are mentioned in the text or identified in an illustration in order to adequately specify the experimental procedure and equipment used. In no case does such identification imply recommendation or endorsement by the National Institute of Standards and Technology, nor does it imply that the products are necessarily the best available for the purpose.
